# Characterization of the rs2802292 SNP identifies *FOXO3A* as a modifier locus predicting cancer risk in patients with PJS and PHTS hamartomatous polyposis syndromes

**DOI:** 10.1186/1471-2407-14-661

**Published:** 2014-09-11

**Authors:** Giovanna Forte, Valentina Grossi, Valentina Celestini, Giuseppe Lucisano, Marco Scardapane, Dora Varvara, Margherita Patruno, Rosanna Bagnulo, Daria Loconte, Laura Giunti, Antonio Petracca, Sabrina Giglio, Maurizio Genuardi, Fabio Pellegrini, Nicoletta Resta, Cristiano Simone

**Affiliations:** Cancer Genetics Laboratory, IRCCS ‘De Bellis’, Castellana Grotte, 70013 Bari, Italy; Division of Medical Genetics, Department of Biomedical Sciences and Human Oncology (DIMO), Università di Bari “Aldo Moro”, Policlinico, Piazza Giulio Cesare 11, 70124 Bari, Italy; National Cancer Institute, IRCCS Oncologico Giovanni Paolo II, 70124 Bari, Italy; Unit of Biostatistics, DCPE, Fondazione Mario Negri Sud, Santa Maria Imbaro, 66030 Chieti, Italy; Medical Genetics Unit, Meyer University Hospital, 50139 Florence, Italy; Dipartimento di Scienze Biomediche, Sperimentali e Cliniche, University of Florence, 50139 Florence, Italy; Institute of Medical Genetics, “A. Gemelli” School of Medicine, Catholic University, 00168 Rome, Italy; Unit of Biostatistics, Scientific Institute Casa Sollievo della Sofferenza, San Giovanni Rotondo, Foggia, 71013 Italy

**Keywords:** Hamartomatous polyposis syndromes, PJS, PHTS, FOXO3A, Cancer risk

## Abstract

**Background:**

Hamartomatous polyposis syndromes (HPS) are inherited conditions associated with high cancer risk. They include the Peutz-Jeghers and the PTEN hamartoma tumor syndromes, which are caused by mutations in the *LKB1* and *PTEN* genes, respectively. Estimation of cancer risk is crucial in order to optimize surveillance, but no prognostic markers are currently available for these conditions. Our study relies on a ‘signal transduction’ hypothesis based on the crosstalk between LKB1/AMPK and PI3K/PTEN/Akt signaling at the level of the tumor suppressor protein FoxO3A. Interestingly, the *FOXO3A* rs2802292 G-allele was shown to be associated with longevity, reduced risk of aging-related diseases and increased expression of FoxO3A mRNA.

**Methods:**

We typed rs2802292 in 150 HPS unrelated patients and characterized the expression of FoxO3A by quantitative PCR and immunoblot analysis in human intestinal cell lines.

**Results:**

We found a significantly higher risk for malignancies in females and TT genotype carriers compared to patients having at least one G-allele. Subgroup analysis for each HPS syndrome revealed a G-allele-associated beneficial effect on cancer risk occurring mainly in males. Molecular characterization of human intestinal cell lines showed that the G-allele significantly correlated with increased basal expression of FoxO3A mRNA and protein.

**Conclusion:**

Our results suggest an inverse correlation between the protective allele (G) copy number and cancer risk, and might be useful to optimize surveillance in HPS patients. Further investigations are needed to confirm our hypothesis and to ascertain whether differences in therapeutic response exist across genotypes.

## Background

Hamartomatous polyposis syndromes (HPS) - Peutz-Jeghers syndrome (PJS), PTEN hamartoma tumor syndrome (PHTS) and juvenile polyposis syndrome (JPS) - are inherited conditions showing hamartomatous polyp histology and increased risk of cancer during lifetime. Hamartomatous polyps originate from uncontrolled proliferation of stromal cells and represent a small fraction of all polyps arising in the GI tract [1].

PJS is an autosomal dominant disease with an estimated prevalence of 1/8,300 to 1/200,000, and is characterized by the presence of mucocutaneous pigmentation, hamartomatous polyps and an increased risk of cancer at different sites (breast, GI tract, gynecological tumors) [2]. PJS is caused by mutations in the *LKB1* tumor suppressor gene, which encodes a serine/threonine kinase [3].

PHTS has a prevalence estimate of 1/200,000 and comprises a group of phenotypically diverse rare autosomal dominant conditions including Cowden syndrome (CS) and Bannayan-Riley Ruvalcaba syndrome (BRRS) [4]. These are caused by germline mutations in the *PTEN* tumor suppressor gene, which encodes a phosphatase. Hamartomatous tumors can affect any organ, namely skin, mucosal membranes, GI tract and other organs in CS, and GI tract in BRRS, which is also associated with macrocephaly, lipomatosis, and pigmented macules of the glans penis. PHTS shows an increased risk of malignancies of the breast, colorectum, thyroid, kidney and endometrium [5].

Several reports estimated cancer risk in HPS. PJS and PHTS patients show a time-dependent high risk of malignancies, with females displaying a significantly higher risk than males mainly due to the occurrence of breast and gynecological tumors [2, 4].

Estimation of cancer risk is crucial in order to implement risk-reducing measures, including intensive surveillance, lifestyle changes, chemoprevention or even prophylactic surgery. However, there is currently no available marker that can predict which HPS patients will develop a malignancy and the age at which surveillance should be started. Recently, genetic modifiers have been shown to play a role in determining cancer risk in other mendelian tumor syndromes, such as BRCA1/2-related breast and ovarian cancer, [6, 7] and SNP genotyping could be of help in identifying a ‘modifier locus’ to predict the risk of cancer in HPS patients.

**Figure 1:**
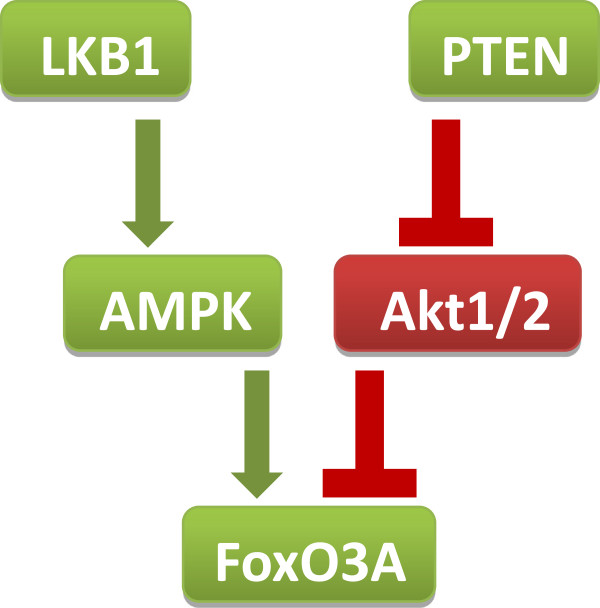
**Our study is based on a ‘signal transduction’ hypothesis, which relies on the crosstalk between LKB1/AMPK and PI3K/PTEN/Akt signaling at the level of FoxO3A.** In particular, LKB1 activates AMPK, which in turn activates FoxO3A, while PTEN inhibits Akt, which in turn inhibits FoxO3A.

Our study is based on a ‘signal transduction’ hypothesis, which relies on the crosstalk between LKB1/AMPK and PI3K/PTEN/Akt signaling at the level of FoxO3A (Figure 1). In particular, LKB1 activates AMPK, which in turn activates FoxO3A, while PTEN inhibits Akt, which in turn inhibits FoxO3A [8]. Recently, it was found that the *FOXO3A* locus strongly correlates with the longevity phenotype in genetically diverse groups of European and Asian descent [9–13]. Of note, the FoxO3A rs2802292 G-allele (minor allele count/MAF = 0.449/978) [14] was shown to be associated with longevity in all populations tested, [9–13] and its copy number correlated with reduced frequency of aging-related diseases, including cancer, in centenarians [9]. At the molecular level, the rs2802292 G-allele displayed significant correlation with increased basal expression of FoxO3A mRNA in muscle biopsies of twins, suggesting that the second intron of the *FOXO3A* locus, which contains the rs2802292 SNP, may act as a regulatory sequence [15].

These data suggest that the rs2802292 G-allele could enhance the well-known metabolic and anti-aging activities of *FOXO3A* by increasing gene expression. Indeed, FoxO3A plays a role in proliferation/arrest, survival/death, metabolism and autophagy, and has been implicated in tumor suppression, regulation of energy metabolism and development in a number of tissues [8]. All these functions are mediated by the finely tuned activation of a coordinated transcriptional program encompassing genes involved in cell cycle, metabolism, autophagy, stress resistance and cell death [8].

To ascertain whether the positive effect of the rs2802292 G-allele on FoxO3A activity could counteract the detrimental effects of imbalanced AMPK/Akt signals on transformation and cancer progression in PJS and PHTS tissues, we typed this polymorphism in a group of unrelated patients previously characterized for *LKB1* or *PTEN* mutations.

## Methods

### Participants

The FoxO3A rs2802292 SNP was analyzed in 150 HPS unrelated patients with identified mutations in the *PTEN* (84 patients) or *LKB1* (66 patients) genes. *PTEN* or *LKB1* mutation carriers were recruited through various Italian cancer genetics clinics and fulfilled the diagnostic clinical criteria for PJS or PHTS, [16, 17] and/or they were carriers of the familial disease-causing mutation. We obtained participants’ informed consent approved by the local ethical committees (AOU Policlinico, 70124 Bari, Italy; Meyer University Hospital, 50139 Florence, Italy) for publication of the dataset at recruitment into the study in compliance with international and national data protection laws. The dataset is fully anonymous, as it does not contain any direct or indirect identifier, thus respecting participants’ rights to privacy and protecting their identity.

### Cell culture and reagents

HT-29, Caco-2, LS174T, HCT-116 cells (all from ATCC) were grown in DMEM supplemented with 10% FBS (HT-29, LS174T and HCT-116) or 20% FBS (Caco-2), 100 IU/ml penicillin and 100 μg/ml streptomycin in a humidified incubator at 37°C and 5% CO2 avoiding confluence at any time.

### Genotyping

Genomic DNA from peripheral blood and cell lines was extracted using QIAsymphony SP/AS instruments (QIAGEN) according to the manufacturer’s protocol and quantified on a NanoDROP 2000 spectrophotometer (Thermo Scientific). PCRs were carried out in 25 μl reaction mixtures containing 50 ng of genomic DNA, 1X PCR Buffer (Tris–HCl, (NH4)2SO4, 15 mM MgCl2; pH 8.7), 200 μM dNTPs and 0.5 U HotStarTaq DNA Polimerase (QIAGEN) and the following primers (10 pmol each): FoxO3A rs2802292g/t Fw, cagcttctgagtgacagagtg and FoxO3A rs2802292g/t Rw, ttcttccctagagagcagcag. PCR amplification cycles were carried out at 95°C for 15 min followed by 29 cycles of denaturation at 94°C for 1 min, annealing at 60°C for 1 min and extension at 72°C for 1 min, and then a final extension at 72°C for 10 min on a GeneAmp PCR System 9700 thermocycler (Applied Biosystems). 5 μl of the amplified products were loaded onto 2% Agarose Standard Low EEO (AB Analitica) in 0.5X TBE and visualized using GelRedTM (Biotium, Hayward, CA). Sequencing products were purified by use of the DyeEx™ 2.0 Spin Kit (QIAGEN, Milan, Italy) and sequenced on an ABI PRISM 310 Genetic Analyzer (Applied Biosystems).

### Quantitative real time PCR

Total RNAs were extracted using TRI Reagent (Sigma). Samples were treated with DNase-1 (Ambion) and retro-transcribed using the High Capacity DNA Archive Kit (Applied Biosystems). PCRs were carried out using the SYBR Green PCR Master Mix on an ABI 7500HT machine (Applied Biosystems). Relative quantification was done using the ddCT (Pfaffl) method. Primer sequences are available upon request.

### Immunoblotting analysis

Immunoblotting analyses were performed according to Cell Signaling’s instructions. Briefly, cells were homogenized in 1X lysis buffer (50 mM Tris–HCl pH 7.4; 5 mM EDTA; 250 mM NaCl; 0.1% Triton X-100) supplemented with protease and phosphatase inhibitors (1 mM PMSF; 1.5 μM pepstatin A; 2 μM leupeptin; 10 μg/ml aprotinin, 5 mM NaF; 1 mM Na3VO4). 15 to 20 μg of protein extracts from each sample were denatured in 5× Laemmli sample buffer and loaded into an SDS-polyacrylamide gel for western blot analysis. Western blots were performed using anti-β-Actin (Sigma) and anti-FoxO3A (Cell Signaling). Western blots were developed with the ECL-plus chemiluminescence reagent (GE Healthcare) as per manufacturer’s instructions.

### Statistical methods

Patient characteristics were reported as medians and interquartile range (IR), and frequency and percentages, for continuous and categorical variables, respectively. Characteristics were also stratified according to the presence of malignant tumors, mutation type and genotype, and compared using Pearson’s χ^2^ and Mann–Whitney U tests for categorical and continuous variables, respectively. To account for potential confounding, presence of malignant tumors was analysed with multivariate logistic regression models and the following covariates were included: gender, age at diagnosis (in years) and genotype (TT and XG). Results were reported as odds ratios (ORs) along with their 95% confidence intervals (95% CI). Adjusted risks for each variable employed in the models were also estimated. Two-sided p-values < 0.05 were considered statistically significant. All statistical analyses were performed using SAS Statistical Package version 9.3 (SAS Institute, Cary, NC).

## Results

A total of 150 patients were analyzed. Median age at diagnosis was 18 (IR 1–77) years, females were 44.7%, and patients with *LKB1* and *PTEN* mutations were 56% and 44%, respectively. Prevalence of the rs2802292 G-allele was 45.3%, which is consistent with the MAF previously described for the general population [14].

Overall cancer risk for our sample was 19.7%. Patient characteristics according to the presence of malignancies (see Table 1 for cancer locations) are shown in Table 2. Patients with and without cancer tended to differ significantly in terms of gender, age at diagnosis and TT genotype. Results for case-mix (i.e. gender and age at diagnosis) adjusted analyses are provided in Table 3. A significantly higher risk of malignancies was found for females (OR 3.33, 95% CI 1.32-8.33; p: 0.011) and TT genotype carriers compared to patients having at least one G-allele (XG) (OR 2.53, 95% CI 1.01-6.34; p: 0.048). This genotype-associated risk increase was slightly greater in PJS (OR 2.82 95% CI 0.74-10.81; p: 0.128) than in PHTS (OR 2.14 95% CI 0.46-9.93; p: 0.332) (Tables 4 and 5). Furthermore, females carrying at least one G-allele (XG) showed a cancer risk of 28% (95% CI 15-44%), which increased to 35% (95% CI 17-58%) for TT females. Of note, only 6% (95% CI 2-16%) of XG males had cancer, while the percentage rose to 25% (95% CI 12-47%) for TT male carriers. Subgroup analysis for each syndrome revealed that the G-allele-associated beneficial effect on cancer risk occurs mainly in HPS males [PJS males with cancer: XG 7% (95% CI 1-27%) vs TT 22% (95% CI 5-62%); PHTS males with cancer: XG 4% (95% CI 0-27%) vs TT 28% (95% CI 9-59%)]. Of note, PJS females carrying the TT genotype were the subgroup with the highest cancer rate [49% (95% CI 21–77)]. This latter result suggests an inverse correlation between the copy number of the protective allele (G) and the risk of cancer, which would be consistent with the reduced frequency of aging-related diseases, including cancer, observed in centenarians [9].

**Table 1:** **Tumor number and location in PJS and PHTS patients**

	PHTS patients*	PJS patients**
**Thyroid**	4	1
**Breast**	3	4
**Gynecological tract**	2	10
**Kidney**	2	0
**Gastrointestinal tract**	1	3
**CNS**	1	0
**Other**	1	0
**Total**	14	18

*2 patients with multiple tumors.

**3 patients with multiple tumors.

**Table 2:** **Patients characteristics according to the presence of malignant tumors**

		Malignant tumors		
Variable	Category	No	Yes	p-value
n		106	26	
Age		31.00 (3.00-78.00)	48.00 (13.00-85.00)	0.001
Age at diagnosis		17.00 (1.00-72.00)	24.50 (5.00-77.00)	0.0421
Genotype	GG	28 (26.42)	5 (19.23)	0.1062
	TG	48 (45.28)	8 (30.77)	
	TT	30 (28.30)	13 (50.00)	
Genotype (2 levels)	XG	76 (71.70)	13 (50.00)	0.0344
	TT	30 (28.30)	13 (50.00)	
Mutation	*LKB1*	56 (52.83)	16 (61.54)	0.4242
	*PTEN*	50 (47.17)	10 (38.46)	
Sex	F	39 (36.79)	17 (65.38)	0.0082
	M	67 (63.21)	9 (34.62)	
Benign tumors	No	27 (25.47)	3 (11.54)	0.1287
	Yes	79 (74.53)	23 (88.46)	
G.I. benign tumors	No	43 (40.57)	4 (15.38)	0.0163
	Yes	63 (59.43)	22 (84.62)	
G.I. malignant tumors	No	106 (100.00)	21 (80.77)	<0.0001
	Yes	0 (0.00)	5 (19.23)	

Data are expressed as medians and interquartile range, and frequency and percentages, for continuous and categorical variables, respectively. P values refer to Pearson’s χ^2^ and Mann–Whitney U tests for categorical and continuous variables, respectively.

**Table 3:** **Multivariate logistic regression model for the presence of malignant tumors**

Parameter	Label	OR (95% CI)	P value
Sex	M VS F	0.30 (0.12-0.76)	0.0114
Genotype (2 levels)	TT VS XG	2.53 (1.01-6.34)	0.0484
Age at diagnosis		1.02 (0.99-1.04)	0.1797

Model is adjusted for gender, age at diagnosis (in years) and genotype (2 levels).

**Table 4:** **Multivariate logistic regression model for the presence of malignant tumors in PJS patients**

Parameter	Label	OR (95% CI)	P value
Sex	M VS F	0.23 (0.06-0.94)	0.0407
Genotype (2 levels)	TT VS XG	2.82 (0.74-10.71)	0.1285
Age at diagnosis		1.02 (0.97-1.06)	0.4816

The model is adjusted for gender, age at diagnosis (in years) and genotype (2 levels).

**Table 5:** **Multivariate logistic regression model for the presence of malignant tumors in PHTS patients**

Parameter	Label	OR (95% CI)	P value
Sex	M VS F	0.52 (0.11-2.42)	0.4038
Genotype (2 levels)	TT VS XG	2.14 (0.46-9.93)	0.3317
Age at diagnosis		1.02 (0.99-1.06)	0.1917

The model is adjusted for gender, age at diagnosis (in years) and genotype (2 levels).

**Figure 2:**
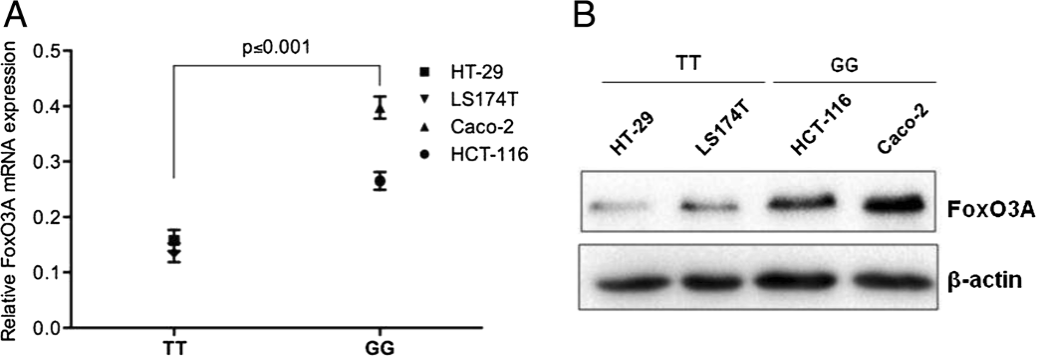
**GG genotype is associated with increased expression of FoxO3A both at the mRNA and protein level.** HT-29, LS174T, HCT-116 and Caco-2 human intestinal cells were typed for the rs2802292 polymorphism and then analyzed by quantitative real-time PCR **(A)** and immunoblotting **(B)** to measure FoxO3A mRNA and protein expression. β-Actin was used as a loading control.

To get insight into the molecular mechanism possibly explaining the beneficial effect of the rs2802292 G-allele, we measured FoxO3A mRNA and protein levels in human intestinal cell lines. Based on our results, cells carrying the GG genotype (HCT-116, Caco-2) showed significantly higher expression of FoxO3A mRNA and protein compared to cells with the TT genotype (HT-29, LS174T) (Figure 2). These data are in agreement with the analysis performed in muscle biopsies of 190 twins indicating that the rs2802292 G-allele was associated with increased basal expression of FoxO3A mRNA [15].

## Discussion

None of the other parameters (mutation type, presence of benign tumors and age at diagnosis) significantly influenced cancer risk in our cohort. These results are of high interest because various research groups throughout the world reported cancer risk estimates of up to and over 80% for HPS patients by age 70 [2, 4]. Based on our data, we speculate that HPS subjects carrying a GG genotype (which supposedly make up around 20% of the overall HPS population) are less likely to develop cancer or tend to be affected at an advanced age, while TT subjects develop malignancies earlier in life and TG individuals show an intermediate phenotype. Indeed, our ‘signal transduction’ hypothesis, which proposed FoxO3A as the crossroad of the HPS pathways LKB1/AMPK and PI3K/PTEN/Akt (Figure 1), is supported by our finding that the GG genotype is associated with increased expression of FoxO3A both at the mRNA and protein level (Figure 2). Regulation of FoxO3A protein expression and localization is crucial for cancer progression and treatment. Indeed, FoxO3A is downregulated in several neoplasms, [18] and inducing increased FoxO3A expression levels is often sufficient to trigger its transcriptional program in cancer cells leading to cell cycle arrest, metabolic regulation and cell death [19].

## Conclusions

Given the relatively small sample size and the cross-sectional design of the study, we cannot exclude the possibility of uncontrolled biases and residual confounding, which is why this hypothesis needs to be confirmed on a higher number of patients and on different populations, as well as through prospective studies. These investigations will also be crucial to ascertain whether differences exist in terms of therapeutic response across genotypes and if mortality is influenced by the rs2802292 allele in HPS subjects. Indeed, FoxO3A, which is a well-known tumor suppressor gene, has emerged as a key downstream effector of various drugs used in tumor treatments, such as p38 inhibitors, cisplatin, paclitaxel, doxorubicin, imatinib, PI3K-Akt inhibitors, EGFR/HER2 inhibitors, and ionizing radiation [8].

## Abbreviations

HPS: Hamartomatous polyposis syndromes
PJS: Peutz-Jeghers syndrome
PHTS: PTEN hamartoma tumor syndrome
JPS: juvenile polyposis syndrome
CS: Cowden syndrome
BRRS: Bannayan-Riley Ruvalcaba syndrome.
